# Dietary Trace Elements and Bioactive Compounds in Food: Exploring Their Health Implications

**DOI:** 10.3390/foods15193550

**Published:** 2026-10-06

**Authors:** Changhui Zhao

**Affiliations:** College of Food Science and Engineering, Jilin University, 5333 Xi’an Road, Changchun 130062, China; czhao@jlu.edu.cn

## 1. Introduction

Dietary trace elements and food-derived bioactive compounds are indispensable components of human nutrition [1]. Beyond their classical nutritional roles, these constituents sustain metabolic homeostasis, antioxidant defense and immune function, and their intake has been repeatedly associated with the modulation of the risk of multiple chronic diseases [2]. Nevertheless, micronutrient deficiencies remain a major global public health challenge affecting a substantial proportion of the world’s population, while dietary patterns increasingly contribute to the burden of non-communicable diseases such as type 2 diabetes and colorectal cancer [3].

Phytochemicals, trace minerals and plant-origin functional ingredients have attracted growing research interest because of their promising protective effects against colorectal cancer, type 2 diabetes, oxidative damage and inflammatory disorders [4,5]. However, the translation of these encouraging findings into dietary recommendations and functional food development is hindered by several bottlenecks, including the reliable assessment of bioaccessibility and bioavailability, the complex molecular interactions between bioactive compounds and food matrices, and the persistent translational gap between in vitro observations and human-relevant health outcomes [6,7,8]. Food processing further modulates the content and activity of nutrients and bioactives, adding another layer of complexity that must be considered when evaluating the health implications of foods [9].

Launched to address these research gaps, this Special Issue, entitled “Dietary Trace Elements and Bioactive Compounds in Food: Exploring Their Health Implications”, compiles high-quality original research and review manuscripts. The collected articles span trace-element profiling, bioactive-compound identification, processing-driven nutrient alteration, mechanistic investigation and pre-clinical health evaluation across diverse food matrices, from staple cereals and bee pollen to culinary herbs, spices, sprouted grains and isoflavone-based inclusion complexes.

## 2. An Overview of the Published Articles

This Special Issue received seven contributions—five original research articles and two review articles—which are briefly introduced below to spark the reader’s curiosity and compel them to explore the full texts.

The study by Rumora Samarin and collaborators (Contribution 1) provides a comprehensive trace-element and arsenic speciation dataset for rice, one of the most widely consumed staple cereals worldwide. Using inductively coupled plasma mass spectrometry (ICP-MS) and HPLC–ICP-MS, the authors quantified 29 essential and toxic elements as well as the fractions of four arsenic species in 58 rice samples purchased from Croatian supermarkets, examining the influence of rice variety, cultivation method and origin. Brown rice contained higher concentrations of essential trace elements (Se, Zn, Cu, Fe, Mn, Co and Cr) than white rice. Overall, no general superiority in essential element content was observed for organically cultivated rice relative to their conventionally grown counterparts. The average total arsenic concentration across all samples was 142 ± 57 µg/kg; among rice types, parboiled rice exhibited the highest mean total arsenic levels, and arsenite was the predominant arsenic species. By highlighting the complex relationship between nutritional gains and toxic-element exposure, this work emphasizes the need to balance the health benefits and safety risks of cereal foods when formulating dietary guidance.

The contribution by Sakhraoui and collaborators (Contribution 2) characterizes the nutritional and phytochemical quality of seven multifloral bee pollen samples collected from geographically and botanically distinct regions of Tunisia. Using standardized colorimetric assays, UV–Vis spectrophotometry, high-performance liquid chromatography (HPLC), gas chromatography–mass spectrometry (GC–MS) and atomic absorption spectroscopy, the authors profiled amino acids, sugars, fatty acids, minerals, phenolic compounds, carotenoids and chlorophylls. Amino-acid profiles were relatively similar across samples, although significant quantitative differences (*p* < 0.05) were observed between individual samples; the proportion of total essential amino acids to total amino acids met the nutritional recommendations of the FAO. Sixteen fatty acids were quantified, with α-linolenic, palmitic and oleic acids predominating, and essential minerals such as K, Ca, P, Mg, Zn, Fe, Mn and Cu were present in significant amounts. Despite notable regional variability, Tunisian bee pollen was evaluated overall as a valuable dietary supplement, supporting its use in nutrition-oriented product development.

Han and collaborators (Contribution 3) address the trade-off between processing and nutritional quality in foxtail millet using comparative metabolomics. Compared with whole grains, manually and ultra-polished millets down-regulated 306 to 720 metabolites, including most antioxidants, essential amino acids, fatty acids and vitamins, whereas germination comprehensively activated metabolic activities. Early stages accumulated basic nutrients such as free and functional amino acids, small sugars, and essential fatty acids, the 16 h stage enriched secondary antioxidant metabolites such as flavonoids, and 24 h germination generated more functional components such as sulfur-containing metabolites. Freeze-drying preserved more basic nutrients in sprouted grains than hot-air drying, which instead favored antioxidant accumulation. These findings provide a systematic reference for developing functional millet products with minimized processing loss.

The study by Hontman and collaborators (Contribution 4) establishes the volatile fingerprint of six culinary herbs and six spices using headspace solid-phase microextraction combined with gas chromatography–mass spectrometry (HS-SPME/GC-MS) and explores their bioactivities through complementary in silico and in vitro approaches. A total of 121 volatile organic metabolites were identified, dominated overall by monoterpenoids (48.3%), sesquiterpenoids (14.0%), esters (11.9%) and carbonyl compounds (8.8%) across all analyzed samples. Molecular docking revealed that cuminaldehyde, β-caryophyllene, γ-curcumene, germacrene D and τ-cadinol exhibited high binding affinities towards anti-Alzheimer’s (acetylcholinesterase, butyrylcholinesterase), antioxidant (monoamine oxidase B, inducible nitric oxide synthase) and anti-inflammatory (5-lipoxygenase, cyclooxygenase-2) targets, while Jamaica pepper showed the highest antioxidant and anti-inflammatory activities among the extracts. These findings highlight the promising potential of culinary herbs and spices in modulating inflammation-related processes, although clinical studies are recommended to validate these effects.

Zhang and collaborators (Contribution 5) employed surface-enhanced Raman spectroscopy (SERS) and in silico simulations to dissect the molecular mechanism by which a γ-cyclodextrin/genistein inclusion complex (γ-CD/GEN) catalyzes GPx4-mediated peroxide reduction. The incorporation of γ-cyclodextrin effectively disrupted the aggregated state of genistein, achieving an encapsulation efficiency exceeding 40%. SERS combined with isotope-effect experiments revealed that the reduction of the organic peroxide cumene hydroperoxide depends on both the concentrations of γ-CD/GEN and GPx4 and relies more on radical-initiated reactions, whereas the reduction of hydrogen peroxide is primarily regulated by the concentration of γ-CD/GEN and involves proton transfer. Molecular docking and molecular dynamics simulations confirmed that γ-CD/GEN stably binds to the Sec(Cys)-46 site in the active center of GPx4, thereby enhancing its catalytic activity. Cellular experiments further demonstrated that γ-CD/GEN elevated GPx activity in CHP-damaged cells and mitochondria; for H_2_O_2_ induced injury, enhanced GPx activity was observed only in mitochondria rather than whole cells. This work provides a theoretical basis for antioxidant strategies based on the precise regulation of enzyme activity by food-derived bioactives.

The review by Wan and collaborators (Contribution 6) synthesizes the emerging evidence on food bioactives that modulate microRNA (miRNA) profiles for the management of colorectal cancer (CRC), the third-most common cancer worldwide. The authors summarize how typical food components, including curcumin, resveratrol, tea polyphenols and calycosin, regulate specific miRNAs and thereby influence CRC cell proliferation, apoptosis, and drug sensitivity. By linking dietary intervention with epigenetic post-transcriptional regulation, this review proposes new directions for CRC prevention and adjunct therapy, and calls for further dissection of underlying miRNA–target regulatory networks together with clinical validation to support robust diet-based anti-CRC strategies.

The final contribution, a systematic review by Thaiudom and collaborators (Contribution 7), evaluates the efficacy of plant-based interventions in improving glycemic control, insulin sensitivity and lipid profiles in individuals with type 2 diabetes. Based on 26 randomized controlled trials retrieved from PubMed, Scopus and ScienceDirect, the authors found that specific interventions, such as Ziziphus jujuba juice, black tea, caper fruit extract and balanced diets, were associated with positive outcomes; however, the results were inconsistent across different interventions and study quality varied. The review therefore concludes that the evidence remains inconclusive and calls for further high-quality randomized controlled trials to establish the optimal dosage, duration and combinations of plant-based interventions, while highlighting their potential role in supporting self-management for individuals with type 2 diabetes.

## 3. Conclusions

The contributions gathered in this Special Issue, “Dietary Trace Elements and Bioactive Compounds in Food: Exploring Their Health Implications”, illustrate the diversity of analytical, mechanistic and evidence-synthesis approaches currently applied to understand the health implications of dietary trace elements and bioactive compounds. From element and arsenic speciation in staple cereals, the comprehensive nutritional characterization of bee pollen and the processing-driven metabolic changes in millet, to the volatile profiling of culinary herbs and spices, the enzyme-level mechanism of an isoflavone inclusion complex, and the evidence-based evaluation of plant-based diets for chronic disease management, these works collectively advance our understanding of how food-borne constituents influence human health. Together, they reinforce the importance of bridging food analysis, processing science, mechanistic nutrition and clinical evidence to translate scientific insights into safer and healthier food systems. We hope this collection will stimulate further research on bioaccessibility assessment, food matrix interactions and human intervention trials, which remain the key to unlocking the full health potential of dietary trace elements and bioactive compounds.
